# SARS-CoV-2 Infection: A Role for S1P/S1P Receptor Signaling in the Nervous System?

**DOI:** 10.3390/ijms21186773

**Published:** 2020-09-15

**Authors:** Elisabetta Meacci, Mercedes Garcia-Gil, Federica Pierucci

**Affiliations:** 1 Department of Experimental and Clinical Biomedical Sciences “Mario Serio”, University of Firenze, Viale GB Morgagni 50, 50134 Firenze, Italy; federica.pierucci@unifi.it; 2 Interuniversity Institute of Myology, University of Firenze, 50134 Firenze, Italy; 3 Unit of Physiology, Department of Biology, University of Pisa, via S. Zeno 31, 56127 Pisa, Italy; mercedes.garcia@unipi.it; 4 Interdepartmental Research Center “Nutraceuticals and Food for Health”, University of Pisa, 56127 Pisa, Italy

**Keywords:** SARS-CoV-2, COVID-19, sphingosine 1-phosphate, sphingosine 1-phosphate receptors, ACE2, olfactory epithelium

## Abstract

The recent coronavirus disease (COVID-19) is still spreading worldwide. The severe acute respiratory syndrome coronavirus-2 (SARS-CoV-2), the virus responsible for COVID-19, binds to its receptor angiotensin-converting enzyme 2 (ACE2), and replicates within the cells of the nasal cavity, then spreads along the airway tracts, causing mild clinical manifestations, and, in a majority of patients, a persisting loss of smell. In some individuals, SARS-CoV-2 reaches and infects several organs, including the lung, leading to severe pulmonary disease. SARS-CoV-2 induces neurological symptoms, likely contributing to morbidity and mortality through unknown mechanisms. Sphingosine 1-phosphate (S1P) is a bioactive sphingolipid with pleiotropic properties and functions in many tissues, including the nervous system. S1P regulates neurogenesis and inflammation and it is implicated in multiple sclerosis (MS). Notably, Fingolimod (FTY720), a modulator of S1P receptors, has been approved for the treatment of MS and is being tested for COVID-19. Here, we discuss the putative role of S1P on viral infection and in the modulation of inflammation and survival in the stem cell niche of the olfactory epithelium. This could help to design therapeutic strategies based on S1P-mediated signaling to limit or overcome the host–virus interaction, virus propagation and the pathogenesis and complications involving the nervous system.

## 1. Introduction

Severe acute respiratory syndrome coronavirus 2 (SARS-CoV-2), a positive-sense single-stranded RNA virus, has been included by the *Coronaviridae* Study Group (CSG) of the International Committee on Taxonomy of Viruses, into the family of *Coronaviridae* SARS-CoV-2, causes the pandemic coronavirus disease (COVID-19) started in China at the end of 2019.

The most prevalent symptoms of COVID-19 are cough, fever, tiredness, breathlessness, conjunctivitis and, in the severe cases, acute respiratory distress syndrome characterized by diffuse damage of alveolar cells [1]. SARS-CoV-2 infection can harm many other organs, such as liver, kidney, heart and intestine and it can also impair coagulation [2]. Moreover, patients reported olfactory and gustatory dysfunctions [3].

The persistent loss (more than two weeks) of smell after the acute phase of viral infection occurs in approximately 55–80% of European patients [3,4], a rare event when compared to the olfactory dysfunctions caused by influenza, rhinovirus, and other coronavirus infections [5]. The deficit of smell may due to damage of olfactory epithelium and/or to the olfactory pathways to the brain. Damaging the supporting/sustentacular cells, SARS-CoV-2 can compromise the functionality of stem cell niche in the olfactory epithelium, thus limiting epithelial cell replacement and tissue regeneration.

Notably, COVID-19 patients showed neurological signs, such as headache, nausea and vomiting [6,7], stroke, isolated cases of Guillain–Barré syndrome [8] and demyelination [9]. It is unclear whether the neurological symptoms are the result of a direct infection of neurons (via a transynaptical transport to the peripheral or central nervous system (CNS) or of an indirect infection (i.e., of the endothelial cells of the blood-brain-barrier, and/or of inflammatory cells, which reach the CNS). In fact, other Coronavirus, such as human CoV-OC43, are able to enter human epithelial and neuronal cells and the mouse CNS [10,11]. Studies in transgenic animal models for the SARS-CoV receptor ACE2 demonstrate the ability of SARS-CoV to reach the brain via the olfactory bulb, leading to transneuronal diffusion to areas of the brain [12]. SARS-CoV-2 has been detected in the cerebrospinal fluid of a patient affected by multiple sclerosis (MS) [13] and in the frontal cortex of an autoptic specimen [14]. In contrast, a recent large screening for SARS-CoV-2 in the cerebrospinal fluid (CSF) of COVID-19 patients reported only two positive out of 578 samples [15].

In response to SARS-CoV infection and/or secondary infections, the immune cells are recruited to the damaged/infected tissue from the circulation and the vast release of cytokines by these cells can result in a “cytokine release storm” and symptoms of sepsis. In several patients, the excessive immune response has been positively correlated with the severity of COVID-19 disease [2]. The inflammatory response involves the synthesis and release of cytokines, chemokines, extracellular matrix proteins, and various lipid factors, including sphingolipid (SL) metabolites.

**Figure 1:**
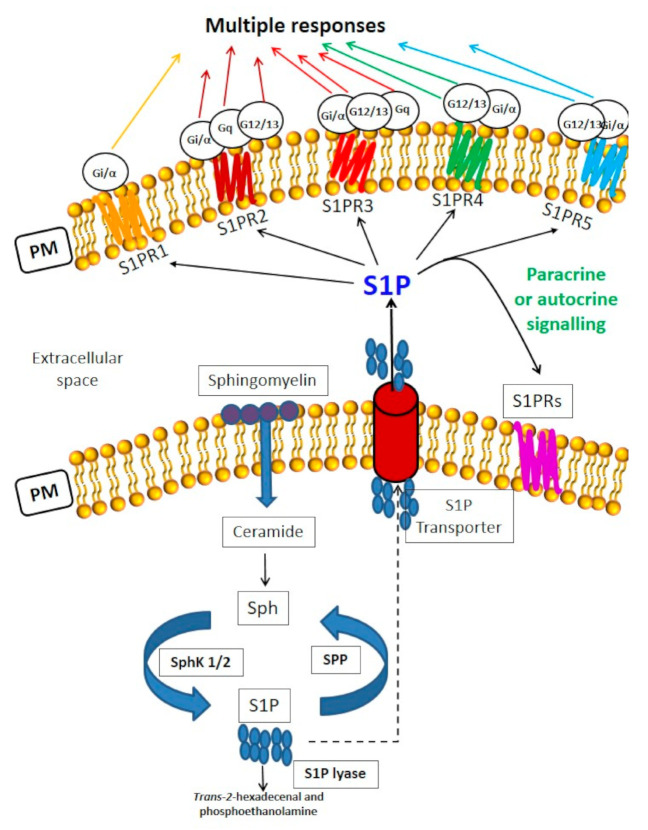
Sphingosine 1-phosphate-mediated signaling and sphingolipid metabolism. S1P plays a role as an intracellular mediator as well as a ligand of specific heterotrimeric GTP binding protein-coupled receptors, S1PRs, which determine multiple responses.S1P transport outside the cells is mediated by specific S1P transporters belonging to the Major Facilitator Superfamily, such as Spinster 2 (Spns 2) and Mfsd2b. *Sphingolipid metabolism*. Sphingomyelin is the more concentrated SL in the plasma membrane (PM) of mammalian cells. It can be hydrolysed by sphingomyelinases generating phosphocholine and ceramide [17,21]. The *de novo* synthesis (not shown) of SLs initiates from serine and palmitate condensation catalysed by serine palmitoyltransferase. The product, 3-keto-dihydrosphingosine, is first reduced and acylated to dihydroceramide, and then reduced to ceramide. *De novo* ceramide synthesis occurs in the endoplasmic reticulum Ceramide can also be phosphorylated to ceramide 1-phosphate (C1P) and substrate of ceramidase, producing sphingosine (Sph). Subsequent phosphorylation of Sph by two SphK isoforms (SphK1/2) generates the bioactive lipid S1P. Sphingosine-1-phosphate phosphatase (SPP) and lipid phosphate phosphatases cause S1P dephosphorylation to Sph, whereas S1P lyase degrades it to hexadecenal and ethanolamine phosphate. Notably, the interplay and the ratio between S1P and its precursors, in particular, ceramide and Sph, is a crucial determinant of cellular fate. Ceramide and Sph can regulate cell growth arrest, senescence and death, whereas S1P and C1P control cell proliferation and survival.

Sphingolipids (SLs) are a large group of bioactive signaling molecules that are critical in many physiological and pathological conditions, including viral infection [16,17,18,19]. The interplay and the ratio between the most studied bioactive SLs, sphingosine 1-phosphate (S1P) and its metabolic precursors, in particular, ceramide and sphingosine (Sph), are crucial in determining the cellular fate (Figure 1) [20]. Interestingly, S1P can play a role as an intracellular mediator as well as a receptor-ligand, which binds to specific heterotrimeric guanosine-5′-triphosphate (GTP) binding protein-coupled receptors, named S1PR1-5 [21,22]. S1P can be released from the cells into the extracellular milieu.

A large family of ATP-Binding Cassette (ABC) Transporters has been firstly identified as S1P transporter in various types of normal and tumoral cells [23,24]. More recently, a member of the MFS (Major Facilitator Superfamily) named Spinster (Spns 2), without a typical ATP binding motif, was identified to contribute to S1P export mainly in heart and in the brain. Spns 2 is also expressed in blood endothelial cells, erythrocytes, platelets, skeletal muscle and skin [25]. Another specific S1P transporter is Mfsd2b (Major Facilitator Superfamily domain-containing 2b) expressed in the brain, bronchial epithelial cells, placenta, bone marrow, red blood cells and platelets. Mfsd2b is essential for the export of the bioactive lipid from endothelial cells in the brain [26]. To date, no data have shown the expression of S1P transporter at the protein level in the olfactory system.

Alfonso et al. (2015) [27] have suggested that activation of S1P1R has a role in the migration of the newborn neuroblasts from the neurogenic niche to the olfactory bulb. S1P treatment appeared able to enhance the proliferation of primary cultured olfactory glial-like cells via S1PR1, supporting a role for S1P in stem cell proliferation and function of olfactory sensory neurons [28]. Interestingly, the olfactory epithelium cells possess high S1P lyase expression and activity [29]. S1PRs show distinct tissue expression [21,22,30] and, recently, it has been reported that they are highly expressed in the olfactory epithelium in vitro as well as in vivo [28].

In many circumstances, S1P has been described as a crucial inflammation regulator [22]. The immunoregulatory effect of Fingolimod (FTY720), a modulator of S1PR, approved for the treatment of multiple sclerosis (MS) [31], might be protective in the case of a cytokine storm. Lipid species are involved in different steps of the viral life cycle and cell host infection. Indeed, several drugs affecting lipid metabolism have been proposed for antiviral therapy, such as statin for the hepatitis C virus [32] and also COVID-19 treatment [33].

Several studies indicate that Fingolimod (FTY720) could be used for the treatment against human immunodeficiency virus (HIV) remission affecting the viral persistence and T cells functions ([34,35]. Regarding SARS-CoV-2, it has been described as a case of a COVID-19 patient under Fingolimod (FTY720) treatment for MS (a 57-year-old female) with a positive outcome [36], thus indicating that the immunomodulation might be beneficial to reduce mortality. However, another patient with relapsing-remitting MS showed reactivation of COVID-19 with signs of hyperinflammation syndrome after Fingolimod (FTY720) withdrawal, indicating that the timing of drug administration may be crucial [37]. A clinical trial with Fingolimod (FTY720) in 30 COVID-19 patients is undergoing (NCT04280588).

It is plausible that not only the signaling triggered by S1P but also the enzymes involved in S1P synthesis or degradation could be impaired by SARS-CoV-2 infection, affecting the viral ability to promote multiple clinical symptoms and the individual response to the virus.

The aim of the present review is to present the properties of S1P that are particularly interesting, spreading from the regulation of virus infection/replication to cell survival, stem cell proliferation and inflammation. In addition, exploring the effects of S1P in the olfactory epithelium as well as in sensorial pathways may open new windows on the therapeutic opportunities against the neurological symptoms and the excessive immune responses that characterize this novel, overwhelming and worldwide infection.

## 2. SARS-CoV-2 Infection

SARS-CoV-2 belongs to the subgenus *Sarbecovirus* of the genus Betacoronavirus. Like other coronaviruses, each SARS-CoV-2virion has the viral envelope composed by structural proteins, including protein S (Spike), E (envelope), M (membrane) and protein N (Nucleocapsid), which holds the RNA genome. Phylogenetic analysis on the entire genome suggests that the virus shares more than 70% similarity to a group of SARS-like coronavirus, and that is likely recombinant between the coronavirus expressed in bat and other genotypes with unknown origin [38,39,40]. Through next-generation sequencing of broncho-alveolar lavage fluid samples from patients infected by SARS-CoV-2, Lu and others [41] have demonstrated that the sequences of SARS-CoV-2, from different infected individuals, are very similar, with greater than 99.9% sequence homology [41]. Contrarily, the S1 domain of the envelope spike protein, responsible for receptor binding, had only around 68% identity with other bat-derived viruses. Individuals can be infected by SARS-CoV-2 through the small droplets, emitted through a sneeze or cough by asymptomatic people or by infected patients and by the contact with contaminated objects or surfaces and, then, touching their eyes, nose or mouth [42].

Based on the cells infected by the virus, several clinical stages of SARS-CoV-2 infection can be recognized: (a) in the early stage of infection, the inhaled SARS-CoV-2 starts replicating in the nasal cavity, viral burden may be low and the individuals might be infectious and asymptomatic [43]; (b) then, in the majority of infected patients, the virus spreads along the upper airway tracts, causing clinical mild and limited manifestations [39]; (c) then, in a minority of individuals, SARS-CoV-2 reaches the lung and affects preferentially alveolar type II cells, thus leading to severe disease [39] or might enter the brain via the olfactory bulb as demonstrated for others SARS-CoVs [12].

SARS-CoV2 entry into host cells requires S protein processing by the transmembrane serine protease 2 (TMPRSS2) and its binding to the angiotensin converting enzyme II (ACE2) receptor [43,44,45]. SARS-CoV-2 does not utilize other coronavirus receptors, such as aminopeptidase N and dipeptidyl peptidase 4 [38]. Upper human airway epithelial cells are not the only ones that co-express ACE2 and TMPRSS2 [46]. Indeed, ACE2 is also present in olfactory neuroepithelia, in particular, in olfactory horizontal basal cells, microvillar cells and in Bowman’s glands [47,48]. Moreover, ACE2 expression is higher in sustentacular cells compared to the others [47,48,49]. Although olfactory receptor mature neurons do not present ACE2 (or only very low levels) [49], it is possible that SARS-CoV-2 enters and damages the sustentacular and stem cells in the nasal epithelium, which are required for normal olfactory functions and/or regeneration of damaged neurons. The protease TMPRSS2 transcripts are expressed in the sustentacular cells and in lower layers of the olfactory epithelium, including immature olfactory receptor neurons and stem cells, and at a very low level, in mature olfactory receptor neurons [47,49,50]. The co-expression of ACE2 and TMPRSS2 and their higher level in sustentacular cells [49] make them more susceptible to SARS-CoV-2 infection. In addition, no cell types expressing both ACE2 and TMPRSS2 were found in the CNS [12,48].

## 3. Olfactory Epithelium, Olfactory Sensory Neurons, Sustentacular Cells

**Figure 2:**
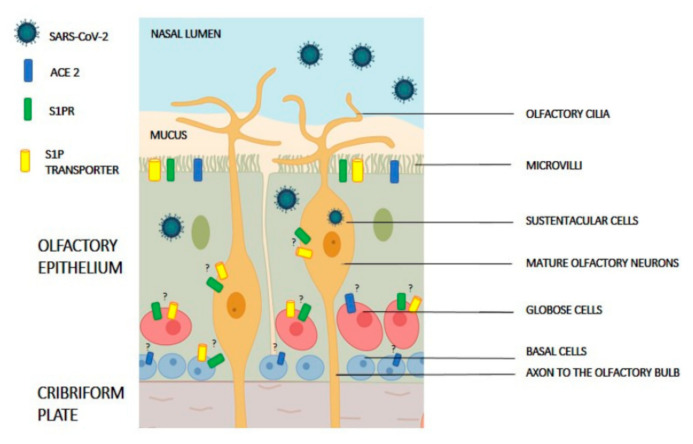
Schematic organization of olfactory epithelium. Known major cell types are shown: non-neuronal cells (progenitor and sustentacular cells) and olfactory neurons. The expression of angiotensin-converting enzyme 2 (ACE2) receptor, sphingosine 1-phosphate receptor (S1PR), S1P transporter (i.e., Spns2 orMfsd2b) and TMPRSS2 are shown in supporting cells as well as neuronal cells. Question marks indicate unknown expression in specific cells.

The olfactory dysfunction can occur by traumatic injury, inhalation of toxic fumes and pathologies, such as cancer and neurodegenerative diseases [51,52]. However, the most common causes of the total (anosmia) or partial (hyposmia) olfactory dysfunctions are the infections, damaging the olfactory epithelium, or to the central olfactory system after viral invasion to the brain [53]. Inflammation of the olfactory mucosa seems to be responsible for the transient olfactory loss associated to the common cold [54]. The olfactory epithelium is a pseudostratified epithelium consisting of sensory neurons, supporting cells, and basal cells, which are stem cells (Figure 2) [51].

The supporting cells of the olfactory epithelium are the glia-like sustentacular cells, located in apical position, and microvillar cells. In particular, these cells display tight junctions with each other and extend long microvilli close to the cilia of sensory neurons [55]. The supporting cells provide essential metabolic and physical support for the olfactory neurons [51] and are involved in the elimination of dead neurons. In fact, after bulbectomy in newborn mice, transmission electron microscopy observations indicate the presence of phagocytizing apoptotic bodies within the supporting cells [56]. Moreover, it is well documented the role of growth factors, feedback signals, such as ActivinB and BMP-4, together with Notch-Delta signals released from the sustentacular cells to the neighbouring cells, resulting in cell survival, proliferation and quiescence [51].

Damaged cells may also release inflammatory factors leading to altered microenvironment and neuron impairment. Injured astrocytes can release inflammatory modulators, chemokines and cytokines (neuroprotective and neurotoxic) and various neurotrophic factors [57].

Single-cell RNA-Seqdataset and database analysis, such as GEO and MGI [47], and, later, immunochemistry and in-situ hybridization experiments, suggest that sustentacular cells express most of the ACE2 and TMPRSS2 [49].

All together, these considerations may support the hypothesis that SARS-CoV-2 infection of sustentacular cells may cause olfactory dysfunctions. Although a small number of these cells may be the target per se of SARS-CoV, the reduced trophic support due to the damaged sustentacular cells infected by SARS-CoV-2 may lead to the impairment in basal cell renewal, thus justifying the observed long-lasting anosmia.

Basal cells are responsible for the continuous regeneration of olfactory epithelium after injury and throughout life. Differently from most regions of the adult nervous system, the stem cell niche in the olfactory epithelium has the capability to regenerate and replace senescent/damaged cells. This olfactory epithelium basal cells are able to differentiate into sensory neurons, microvillar and sustentacular cells. Indeed, olfactory nerve damage and degeneration of olfactory neurons lead to an intense mitotic activity of these basal cells [58] and, consequently, to the recovery of the population of olfactory neurons [51]. Moreover, basal cell differentiation into neurons re-establishes the functional connections to the olfactory bulb.

Exposure of the olfactory epithelium to toxins and chemicals determines mucosal surface degeneration, without any alterations to the deeper layer of basal cells, which are in charge of restoring tissue structure and function providing new sensory and supporting cells [59]. Contrarily, insufficient basal cell functioning leads to the formation of scar tissue that prevents or blocks axons growing [60].

Two distinct stem cell populations exist in the niche: (1) the globose basal cells, which are both reserve cells and progenitors and (2) the reserve horizontal basal cells, which are generally quiescent, but mitotically active after tissue injury [51]. In mammals, the globose basal cells proliferate, differentiate and activate the horizontal basal cells to participate to the reconstitution of the olfactory epithelium [51,61]. Many external stimuli, including factors released from the neighbouring cells, involved in the regulation of olfactory neurogenesis, can be important clinical/drug targets [62].

## 4. SARS-CoV-2 and Inflammation

### 4.1. SARS-CoV-2 and Cytokine Storm

The first line of defense against viral infection is performed by a coordinated innate immune response. However, in many cases, a cytokine storm occurs, leading to an excessive immune response, which progresses rapidly and results in high mortality. During the COVID-19 pandemic, elevated levels of interleukins (i.e., IL-2, IL-6), Granulocyte colony-stimulating factor (G-CSF), Granulocyte-Macrophage Colony-Stimulating Factor GM-CSF, Interferon γ (IFNγ) and Tumor Necrosis Factor α (TNF α) in serum have been reported [1,63,64] accompanied by an increase in naïve helper T-cells and reduction in memory helper and regulatory T-cells [65,66]. Moreover, the serum levels of IL-2 receptor, IL-6,G-CSF,TNF-α and chemokines are positively correlated with the severity of the disease [67]. Several authors have also suggested that the morbidity and mortality of SARS-CoV-2 may be the consequence of these excessive immune reactions and cytokine storms [64,66].

### 4.2. Therapeutical Approaches

A number of drugs with antiviral properties have been proposed as therapy for SARS-CoV-2 and have been recently reviewed by Ye et al. [64]. They include protease inhibitors (lopinavir/ritonavir), previously administered to HIV patients; a nucleotide analogue active against a wide array of RNA viruses (Remdesivir), an RNA polymerase inhibitor, Favipiravir, previously used with Ebola virus-infected patients, and the antimalarial chloroquine [64]. Other therapies address the cytokine storm: Adalimumab is a TNF-α inhibitor undergoing evaluation in a trial registered in China (ChiCTR2000030089), Tocilizumab, an IL-6 antagonist, currently used in rheumatoid arthritis [68,69].

Clinical trials, including Tocilizumab, Sarilumab, a monoclonal antibody that binds the IL-6 receptor, as well as Anakinra, IL-1 receptor antagonist, have been performed (NCT04331808, NCT04324073, and NCT04341584, respectively) [64].

In addition, mesenchymal stem cell (MSC) therapy appears a promising approach. In fact, MSCs not only self-renew and differentiate, but play an anti-inflammatory and immune regulatory functions. MSCs can reduce the aberrant activation of T lymphocytes and macrophages, and the occurrence of cytokine storms [70]. A pilot study involving MSC transplantation to explore their therapeutic potential for COVID-19 patients is being performed (NCT04339660) [62].

## 5. SL Metabolism and the Role of S1P on Inflammation

SLs are both structural and signaling molecules that regulate cell fate and inflammation in physiological and pathological conditions [16,17,18]. SLs are formed by sphingosine (Sph) backbone linked to one hydrophobic acyl chain and a phosphate head group ester.

The metabolism of SLs is described in Figure 1. Some sphingosine or S1P analogues, have been approved for the treatment of MS, and some have entered clinical trials for different diseases, including MS and amyotrophic lateral sclerosis [18,31]. In particular, the structure of Fingolimod (FTY720) is analogous to that of Sph, and it is phosphorylated to Fingolimod (FTY720)-phosphate, a structural analogue of S1P in vivo by SphK2. Fingolimod (FTY720)-phosphate acts as an agonist of S1PRs, except S1PR2, and determines the irreversible internalization and degradation of bound receptors, thereby functioning as receptor antagonist [22,31] (Figure 1).

Fingolimod (FTY720) acts as an immunosuppressant by preventing lymphocyte egress from the lymph nodes, thereby, limiting autoaggressive lymphocyte migration into the CNS. Other S1P analogues, which show higher S1PR specificity than Fingolimod (FTY720), include siponimod and ozanimod, which have entered phase III clinical trials for MS [22]. S1PR ligands, such as AAL-Ror its phosphate ester, inhibit the T-cell response to influenza virus infection in animal models [71].

Some intracellular targets of S1P are reviewed in [22,72]. One of them is prohibitin2, a highly conserved protein that controls mitochondrial assembly and functions. Another is the tumor necrosis factor receptor-associated factor 2 (TRAF-2), an important component of the nuclear factor-κB (NF-κB) pathway, which is involved in inflammatory gene regulation.

S1P, either acting intracellularly or through S1PR, elicits pro-inflammatory and anti-inflammatory responses [72]. Increased mRNA levels of SphK1/2, S1PR2, and S1P lyase in alveolar macrophages have been observed in chronic obstructive pulmonary disease patients compared to control subjects [73]. Many studies have demonstrated that inflammation signals and IL-6 release induced by lipopolysaccharide are mediated by increased S1P level either by S1P lyase down-regulation or by SphK activation [74].

Reduction of SphK activity has a protective effect against various inflammatory insults, including bacterial sepsis, inflammatory arthritis, colitis, allergic asthma, and anaphylaxis in animal models. By contrast, S1P and S1PRs ameliorate inflammation by maintaining vascular integrity [75]. In a murine model of acute lung tissue damage, intratracheal instillation of lipopolysaccharide increased expression of S1P lyase and reduced S1P concentrations in lung tissue and in plasma [76], while S1P and Fingolimod (FTY720) inhibited vascular permeability and alveolar flooding [77].

### 5.1. S1P and Virus Infection

Several studies have demonstrated that infection by a wide variety of viruses affects S1P metabolism and that modification of SphK expression or activity and modulators of S1PRs are able to modify the cellular responses to viral infection. These studies are summarized in Table 1.

In the context of a viral infection, the first report, linking SL metabolites to cell survival, was provided by Monick et al. [78]. These authors reported that respiratory syncytial virus stimulated within minutes neutral ceramidase and SphK activities in lung epithelial cells, likely leading to the S1P accumulation responsible for the Akt/ERK survival pathways. Accordingly, the inhibition of SphK blocked respiratory syncytial virus-induced activation of these pathways and promoted accelerated cell death. In addition, increased SphK1 activity has been found following infection by human cytomegalovirus, influenza virus A [79,80,81,82,83], measles virus [84] and bovine viral diarrhea virus [81,85]. It has been also found that inhibition of SphK1 impaired viral RNA synthesis and the viral ribonucleoprotein translocation from the nucleus to the cytoplasm [81]. SphK and acid ceramidase inhibitors efficiently inhibited measles virus replication [84]. Similarly, a transient inhibition of SphKs protects influenza A virus-infected mice [83]. All these findings support the essential role for SphK1 activation for the progression of infection of several viruses and the efficacy of SphK inhibitors in preventing the infection.

In contrast, SphK1 activity is decreased in the bovine viral diarrhea and dengue virus infections [86]. Regarding the other isoform of SphK, the nuclear-localized SphK2, it has been shown to be a chikungunya virus host factor assembled with the viral replication complex [87] and to promote influenza A virus replication [83]. This SphK isoform is also able to maintain viral latency and survival of Kaposi’s sarcoma-associated herpesvirus-infected endothelial cells [88].

The sphingosine analogs modulating S1PR1,3-5 (i.e., AAL-R and Fingolimod (FTY720)), have been tested. It has been found that AALR limits T-cell infiltration and decreases pro-inflammatory T-cell responses and lung injury in influenza virus-infected animal models [89], but they are not effective in the infections by simian-human immunodeficiency and lymphocytic choriomeningitis virus [90,91]. The S1PR2 antagonist JTE-013 has been shown to decrease human cytomegalovirus replication [92]. Altogether, these studies suggest a potential role for distinct S1PR signaling in some virus infections.

### 5.2. S1P and Nervous System Inflammation

Regarding inflammation in the nervous system, S1P has been extensively investigated in relation to MS [31] and in other pathologies of the CNS, including stroke and Sandhoff’s disease [93]. Fingolimod (FTY720) alters lymphocyte trafficking through receptor subtype S1PR1, although it might also modulate neuronal and glial functions. The importance of astrocytes was uncovered by experiments performed with mice lacking S1PR1 [94]. Experimental autoimmune encephalomyelitis, an animal model of SM, was attenuated by Fingolimod (FTY720), but the efficacy of the SL analog was not observed in mice lacking S1PR1 on astrocytes [94]. It is well established that S1P activates inflammation in murine astrocytes, which also express S1PR3 and S1PR2. S1PR3 controls the activation of RhoA and induction of cycloxygenase-2, IL-6, and VEGFα mRNA, with some involvement of S1PR2 [95]. S1PRs modulated pro-inflammatory chemokine release in astrocytes and microglial cells [96] and Fingolimod (FTY720) blocked transcription of the chemokines CXCL5/LIX, CXCL10 and monocyte chemoattractant protein 1 in astrocytes and attenuated CXCl7 release from microglia [96]. SphK inhibition attenuates the lipopolysaccharide-induced increase in mRNA levels of all three chemokines. Karunakaran et al. [97] have recently found that in mice, in which the gene coding for S1P lyase type 1 has been deleted, S1P accumulated in brain and determined microglial activation and IL-6 secretion, which is associated with autophagy defects and mediated by the S1P/S1PR2 axis. It is important to note that the role of S1P signaling can be different in non-nervous tissue. For example, S1P accumulation caused by inhibition of the S1P-degrading enzyme SPL in lung epithelium cells promotes disease tolerance in experimental sepsis via S1PR3 [98].

### 5.3. Effects of S1P on Neuronal Niche

S1P is a powerful stimulator of neurogenesis [99,100]. S1PRs show distinct levels of expression in different neural cell types and neural progenitor stem cells (NPSCs) [101,102]. The upregulation of S1PR1 was observed in NPSCs derived from embryonic stem cells [103]. In the presence of activated astrocytes, S1P further potentiates the differentiation of NPSCs, as demonstrated by neurite outgrowth and arborization [99,104]. Notably, neural precursors derived from embryonic stem cells express all five S1P receptor mRNAs, with S1PR2 and S1PR3 being the most abundant mRNAs [104]. S1PR1 has been demonstrated to have a role in the migration of the newborn neuroblasts from the neurogenic niche to the olfactory bulb likely by modulating the expression of NCAM1 and β1 integrin [27] and S1PR2 inhibits NPSC migration to the sites of injury [27,105].

Basic fibroblast growth factor promotes the release of S1P from astrocytes. The extracellular S1P, through autocrine or paracrine mechanisms, activates the proliferation of astrocytes mediated by S1PR [106]. S1PR5 is present in mature oligodendrocytes, where the receptor mediates cell survival and cell process retraction [107]. Higher expression level and activation of S1PR1 contributes to the survival of oligodendrocyte progenitors and induces oligodendroglial differentiation. Fingolimod (FTY720) can pass the blood–brain barrier and target NPSCs [108]. Protective effects by Fingolimod (FTY720) signaling have been reported for astrocytes and oligodendrocytes [94,109]. Moreover, Fingolimod (FTY720) increased the viability and of irradiated NPSCs from the hippocampus [110,111], partially restoring neurogenesis [99], although this result was not reproduced by Metzdorf et al. [112]. In addition, Fingolimod (FTY720) increases proliferation of embryonic neural stem cells, hippocampal neurogenesis and learning and memory in adult mice [113,114].

The role of SphK/S1P in neural stem cells has been reported by Meng et al. [115]. The authors documented the expression of SphK1 in neuron and progenitor cells of the mouse embryo [115]. SphK enhances NPSC proliferation and survival during sensory ganglia development since the complete absence of Sphk1 and Sphk2 genes reduced the number of neurons and progenitor cells in trigeminal and dorsal root ganglia.

Summing up, SphK/S1PRs mediate proliferation, survival, differentiation and migration of neural stem cells, survival and differentiation of oligodendrocyte precursors and survival and proliferation of astrocytes.

## 6. Druggability of SL Metabolism and S1P Signaling for COVID-19

Based on the S1P/S1P signaling role in infection by other viruses, it is necessary to study whether this pathway is involved in SARS-CoV-2 biology and on the cellular responses to the infections. If this is the case, modulation of the specific targets (SphK1, ShpK2, S1P lyase, or S1PRs) through inhibitors, agonist or antagonists could block viral entrance, replication or release or virus-induced inflammatory response.

The immunomodulatory action and the ability of SL analogs, such as Fingolimod (FTY720), to enhance lung endothelial cell integrity and to protect the CNS might be beneficial for COVID-19 patients. However, the risks and the collateral effects of Fingolimod (FTY720) treatment reported to some MS-affected COVID-19 patients, may be a limit for this treatment [21,116]. Newer analogs with expected minor collateral effects start to be available [22].

To date, there are other clinical trials for various pathologies, including inflammatory diseases, either completed or in progress, using drugs that target S1PRs and S1P metabolism. For example, the S1PR ligand AKP11 (ACTRN12617001223325) or BMS-986104 (NCT02211469) are utilized for rheumatoid arthritis (phase II) or Etrasimod/APD334 (NCT04173273) for Crohn’s disease, and the SphK2 inhibitor ABC294640 (NCT02757326) for multiple myeloma. Therefore, it will be worth to investigate and experimentally verify the relevance of S1P-triggered signaling and metabolism in SARS-COV-2 infection in more detail in the nervous system and in other tissues.

## 7. Conclusions and Future Directions

A wide variety of reasons justify in-depth studies on the involvement of the bioactive sphingolipid S1P in the control of cell fate after SARS-CoV-2 infection, in the nervous system, including the olfactory system, especially considering the persisting symptoms.

Although it is not well documented whether the virus enters the brain, SARS-CoV-2 might reach the brain through the axonal transport or other mechanisms, which may involve the infection/release from endothelial cells of the blood–brain barrier, and/or inflammatory cells. Extending the knowledge on the bioactive factors such as S1P, which influence the neural cell survival and are, likely, able to modulate virus entrance to the brain, might provide tools for limiting the virus accessibility and/or its persistence in nervous tissue.

SARS-CoV-2 binds to the ACE2 receptor—its wide distribution throughout the body [117] may justify the multiorgan failure in COVID-19 patients [118]. To date, the signaling pathways that influence the expression of both ACE and TMPRSS2 in distinct tissues are unknown, except in the heart, where ACE2 expression is modulated by S1PR1-triggered signaling [119]. Similar crosstalk between S1PR1 and ACE2 expression may occur in other cell types, including olfactory cells. Thirty-two variants of ACE2 [120], higher ACE2 expression in Asian populations with respect to Caucasian and African-American populations [121] and in the blood of men with respect to women [122] have also been reported. The possibility of crosstalk between ACE2 and S1PR1 or other S1PR subtypes, specifically expressed in different cell types might contribute to extend individual diversity of virus infection response.

Notably, the neurological symptoms reported in COVID-19 patients should be seriously considered, but the mechanism remains obscure since no cell type expressing both ACE2 and TMPRSS2 has been found in the CNS. We hypothesize that SARS-CoV-2 infection might impair the known pro-survival, trophic and neurogenic functions of S1P-S1PR axis [18].

Olfactory cell damage and smell loss observed after SARS-COV-2 infection are likely due to an impaired neurogenesis. S1P is reported to act as a pro-survival factor and an important regulator of stem cell proliferation; therefore, S1P production and its transport outside of the olfactory cells, such as supporting cells, may be essential for basal cell proliferation and/or differentiation. Consequently, it will be worthy of considering the effect of SARS-CoV-2 on S1P generation and release in the microenvironment of olfactory epithelium cells. On the other hand, the modulation of S1P release/content, through S1P transporter (i.e., Spns2), SphK and/or S1P lyase activities, and S1PR-mediated signaling may be important therapeutic approaches for COVID-19 because they might prevent SARS-CoV-2 infection or virus propagation (Figure 2). Altogether, the multiples symptoms reported by COVID-19 patients suggest the need for combined pharmacological, immunological, and biochemical approaches. The cytoprotective and the pleiotropic properties of S1P/S1PR signaling may offer a tool to safeguard many of the tissues that are highly vulnerable in COVID-19 patients.

## Figures and Tables

**Table 1:** Involvement of S1P in virus infection. The table illustrates the effect of viral infection on SphK activity/expression as well as the effects on the virus infection response due to modulations/modifications of the expression/activity of enzymes involved in S1P metabolism or modulators of S1PR. The table reports data from RNA viruses (positive-sense, single-stranded RNA) as SARS-CoV2 (BVDV, DENV, CHIKV, LCMV and HIV), from RNA virus (negative-sense, single-stranded RNA) as IAV and measles virus, and from DNA virus (*). BVDV: bovine viral diarrhea virus; CHIKV Chichungunya virus, DENV: dengue virus type 2; HCMV: human cytomegalovirus; HIV: human immunodeficiency virus; IAV: influenza A virus (H1N1); KSV: Kaposi’s sarcoma-associated herpes virus; LCMV lymphocytic choriomeningitis virus; RSV: respiratory syncytial virus.

Virus	Enzyme/S1PR Modulator	Effect	Ref.
**S1P metabolism**			
RSV infection	↑ SphK ↑ ceramidase	↑ infected lung epithelial cell survival	[78]
HCMV * infection	↑ SphK prot/mRNA	↑ virus replication	[79]
IAV infection	↑ SphK1	↑ RNA virus synthesis	[80,81,82]
	SphK1 overexpression	↑ infection susceptibility	
	S1P lyase overexpression	↓ virus replication	
	↓ SphK	protection of infected mice	[83]
MV infection	↓ SphK	↓ replication	[84]
BVDV infection	↑ SphK	↑ replication	[85]
DENV infection	↓ SphK1	↓ cell survival ↑ replication	[86]
CHIKV infection	↑ SphK2	↑ replication	[87]
KSV * infection	↑ SphK2	maintenance latency	[88]
**Signaling**			
IAV	AAL-R (S1PR1,3-5 modulator)	↓ inflammatory response	[71,89]
HIV	FTY720	↓ circulating T and B cells	[90]
LCMV *	FTY720	no lymphopenia, no effect on replication	[91]
HCMV *	JTE-013 (S1PR2 antagonist)	↓ replication	[92]

## Abbreviations

SARS-CoV-2	Severe acute respiratory syndrome Coronavirus-2
COVID-19	Coronavirus disease
ACE2	Angiotensin-converting enzyme 2
TMPRSS2	Transmembrane serine protease 2
SL	Sphingolipids
S1P	Sphingosine 1-Phoshate
S1PR	Sphingosine 1-Phosphate specific Receptor
GTP	Guanosine Tri-Phosphate
S1P	Sphingosine 1-Phosphate
C1P	Ceramide 1-Phosphate
CNS	Central Nervous System
NPSCs	Neural Progenitor Stem Cells
Sph	Sphingosine
SphK	Sphingosine Kinase
SPP	Sphingosine-1-Phosphate Phosphatase
Spns2	Spinster (S1P transporter)
MS	Multiple Sclerosis.
